# Charge-induced chemical dynamics in glycine probed with time-resolved Auger electron spectroscopy

**DOI:** 10.1063/4.0000165

**Published:** 2022-11-08

**Authors:** David Schwickert, Marco Ruberti, Přemysl Kolorenč, Andreas Przystawik, Slawomir Skruszewicz, Malte Sumfleth, Markus Braune, Lars Bocklage, Luis Carretero, Marie Kristin Czwalinna, Dian Diaman, Stefan Düsterer, Marion Kuhlmann, Steffen Palutke, Ralf Röhlsberger, Juliane Rönsch-Schulenburg, Sven Toleikis, Sergey Usenko, Jens Viefhaus, Anton Vorobiov, Michael Martins, Detlef Kip, Vitali Averbukh, Jon P. Marangos, Tim Laarmann

**Affiliations:** 1 Deutsches Elektronen-Synchrotron DESY, Notkestr. 85, 22607 Hamburg, Germany; 2 Department of Physics, Imperial College London, Prince Consort Road, London SW7 2AZ, United Kingdom; 3 Charles University, Faculty of Mathematics and Physics, V Holesovickach 2, 180 00 Praha 8, Czech Republic; 4 The Hamburg Centre for Ultrafast Imaging CUI, Luruper Chaussee 149, 22761 Hamburg, Germany; 5 Helmholtz Institute Jena, Fröbelstieg 3, 07743 Jena, Germany; 6 Helmholtz Centre for Heavy Ion Research (GSI), Planckstr. 1, 64291 Darmstadt, Germany; 7 Friedrich-Schiller-Universität Jena, Max-Wien-Platz 1, 07743 Jena, Germany; 8 European XFEL GmbH, Holzkoppel 4, 22869 Schenefeld, Germany; 9 Helmholtz-Zentrum Berlin für Materialien und Energie, Albert-Einstein-Straße 15, 12489 Berlin, Germany; 10 Faculty of Electrical Engineering, Helmut Schmidt University, Holstenhofweg 85, 22043 Hamburg, Germany; 11 Department of Physics, University of Hamburg, Luruper Chaussee 149, 22761 Hamburg, Germany

## Abstract

In the present contribution, we use x-rays to monitor charge-induced chemical dynamics in the photoionized amino acid glycine with femtosecond time resolution. The outgoing photoelectron leaves behind the cation in a coherent superposition of quantum mechanical eigenstates. Delayed x-ray pulses track the induced coherence through resonant x-ray absorption that induces Auger decay. Temporal modulation of the Auger electron signal correlated with specific ions is observed, which is governed by the initial electronic coherence and subsequent vibronic coupling to nuclear degrees of freedom. In the time-resolved x-ray absorption measurement, we monitor the time-frequency spectra of the resulting many-body quantum wave packets for a period of 175 fs along different reaction coordinates. Our experiment proves that by measuring specific fragments associated with the glycine dication as a function of the pump-probe delay, one can selectively probe electronic coherences at early times associated with a few distinguishable components of the broad electronic wave packet created initially by the pump pulse in the cation. The corresponding coherent superpositions formed by subsets of electronic eigenstates and evolving along parallel dynamical pathways show different phases and time periods in the range of (−0.3±0.1)π≤ϕ≤(0.1±0.2)π and $18.2_{-1.4}^{+1.7}\leq T\leq 23.9_{-1.1}^{+1.2}$ fs. Furthermore, for long delays, the data allow us to pinpoint the driving vibrational modes of chemical dynamics mediating charge-induced bond cleavage along different reaction coordinates.

## I. INTRODUCTION

Since the pioneering experiments by Weinkauf and Schlag on electron mobility and dissociation in peptide cations,^1^ the interplay between local ionization and molecular reactivity is of considerable interest in many areas of physics, chemistry, biology, and in the materials sciences.^2^ Ultrafast photoionization of a molecule leads to a spatial redistribution of electronic charge, i.e., a time-dependent oscillation of its charge density.^3^ Quantum coherences mediating this process are formed and defined by the coherent superposition of several quantum mechanical eigenstates. Pure electronic quantum wave packet dynamics is usually termed “charge migration,” while dynamics involving nuclear degrees of freedom is referred to as “charge transfer.”^2^ The most stable and experimentally easily detectable coherences are formed by a pure two-state system with the oscillation period of the charge migration being defined by the energy difference between the two states lying within the spectral bandwidth of the coherent radiation pulse. Examples studied so far show timescales of 100 as^4^ to 20 fs.^5^

The correlated motion of electrons leads to the hole localizing at a particular site, where a subsequent photoionization event may result in bond cleavage. Thus, quantum coherences increase the speed and efficiency of electron or hole migration to the reaction centers, but it is also possible for them to decohere due to coupling of electronic with nuclear degrees of freedom during the electronic wave packet propagation.^6–8^ If the coherences are preserved for multiple oscillation periods,^9^ the efficiency of photochemical reactions is increased even further, since it is given additional occasions for the charge-induced reaction with each full period after the initial charge formation. Therefore, it is imperative to precisely control the temporal and spectral phase of the radiation as known from photochemical control protocols using table-top lasers.^10–12^ A prerequisite for any control of electronic and nuclear dynamics toward specific molecular reaction pathways is a detailed analysis of structural properties, when the molecule is driven out of equilibrium and here, in particular, the analysis of the time-dependent electronic structure defining the potential energy landscape in which the nuclei move.^13^ X-ray photoelectron spectroscopy (XPS) is a well-established technique sensitive to the electronic structure. Applied in a time-resolved pump-probe scheme, it allows us to unambiguously monitor electronic coherences and vibronic coupling long before fragmentation sets in, i.e., while the electronic wave packet propagates.

Linking the electronic quantum wave packet motion to charge-induced chemical dynamics in photoionized glycine (Gly) molecules is the goal of the present study. The amino acid is an abundant basic building block of proteins and is part of the recognition sites on cell membranes and enzymes.^14^ Due to its compact nature and tendencies to form hydrogen bonds, it facilitates the coiling of proteins and is, therefore, incorporated frequently in hydrophobic protein helices, where it reduces helix packing voids and sets the orientation of multiple helices in a folded protein complex.^15^ Stand-alone, it is utilized as an inhibiting neurotransmitter in the central nervous system.^16^ In aqueous solution, the molecule may exist as a zwitterion, while in the gas phase, it has its canonical neutral form.^17^ Glycine has also been found in space.^18,19^ The molecular reactivity in the harsh astronomical environments is an important aspect, and, in particular, how isolated molecules interact with ionizing radiation is a key question in astrochemistry.

In order to track the dynamical processes occurring in the glycine cation upon inner-valence ionization by a femtosecond (fs) free-electron laser (FEL) pulse, we set the x-ray FEL probe photon energy in our gas-phase study to a local, element-specific core-shell transition below the carbon K-edge as adopted in Ref. 5 and analogues to Ref. 20, performed at the oxygen K-edge. The selected electronic transition into a spatially extended inner-valence orbital induces Auger electron emission that is detected in coincidence with the generated parent and fragment ions. Thereby, the delayed fs x-ray pulses probe the transient local charge (hole) density at the carbon atoms of the parent ion, while the many-electron configuration evolves with some probability along different selected fragmentation pathways.

The present paper is organized as follows. Section II briefly describes the experimental setup, focusing on the molecular beam preparation, the FEL pulse characteristics and optics as well as on the performance parameters of the applied electron and ion spectrometers. Section III reports the experimental results, beginning with time-resolved Auger electron spectroscopy in Subsection III A. Here, orbital-selective information at early times of the charge-induced chemical dynamics is derived from the coincidence and correlation analysis of the simultaneously recorded electrons and ions generated by the FEL pulses. The x-ray interaction with glycine molecules results in both intact Gly^2+^ parent ions and characteristic fragments. The corresponding photoion–photoion coincidence map is discussed in Subsection III B. A detailed wavelet analysis of the recorded time-dependent electron and ion data is presented in Subsection III C. This final subsection on the experimental results highlights the key observations of the present study, which are time-frequency spectra of different coherent superpositions of electronic states dressed by vibrational excitations along different charge-induced reaction pathways. We conclude with a brief summary and outlook. In the Appendix, useful background information on the continuous wavelet transform is given, which will be used extensively in the analysis of time dependent signals with multiple frequency components and variable amplitudes in Sec. III and Subsection III C, respectively.

## II. EXPERIMENTAL SETUP

An effusive molecular beam of glycine molecules, crossing the FEL beam perpendicularly, was produced using a resistively heated oven design. The crystalline powder was acquired from Sigma-Aldrich with >98.5% purity. The sample reservoir has a 150 mm long stainless steel capillary with a 1 mm outer and 0.5 mm inner diameter attached to deliver the sublimated molecules to the interaction zone. Both the crucible and the capillary have respective thermocouples and heating elements. In this way, the capillary can be prevented from clogging. The temperature-controlled molecular beam source was operated at around 160 °C, which results in a partial gas pressure on the order of $10^{-2}$ mbar at the nozzle. Under these conditions, only two conformers commonly referred to as Gly I and Gly III are expected to be present in the beam at a ratio of ∼ 2:1.^21^ An electrostatic potential is applied to the capillary in order to minimize field inhomogeneities affecting the electron and ion spectrometer performance. The capillary is guarded by a ceramic sheath from electrical contact with the proximal spectrometer electrodes. The charged-particle detection axis is oriented perpendicular to the FEL and to the molecular beam direction. The molecular beam oven is mounted on an XYZ-manipulator, so that the orifice can be steered as close as possible (∼1 mm) to the FEL focus resulting in a target density in the interaction zone of about 900 molecules per mm^3^. Exchangeable noble gas atom beams can be fed from the back of the oven through the reservoir and capillary for the calibration of the magnetic-bottle electron spectrometer (MBES) and the time-of-flight (TOF) ion spectrometer, respectively. The design allows for fast cooldown to facilitate the change from glycine to residual gas or noble gas measurements without changing the geometry in the vicinity of the interaction volume with the FEL beam.

**FIG. 1.**
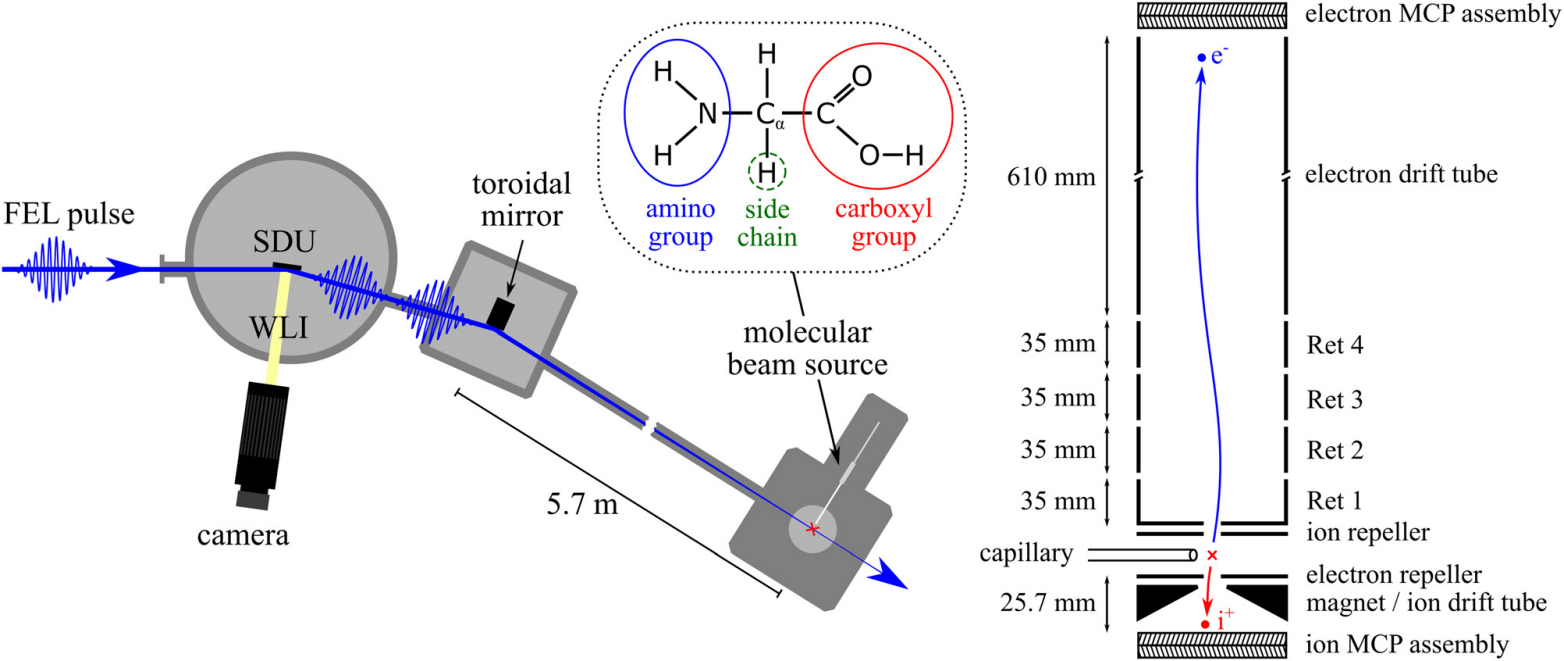
(Left) Top-view of the experimental setup scheme. The FEL pulses are split into two pulse replicas with adjustable time delay by the “Split-and-Delay Unit” (SDU) and focused toward the interaction zone, where the glycine sample is evaporated by a molecular beam source. Absolute calibration of the FEL pump—FEL probe delays is achieved by means of optical laser and white-light interferometry (WLI) tracking the SDU optics displacement. (Right) Cross section of the magnetic bottle electron spectrometer (MBES) and ion time-of-flight (TOF) spectrometer. (Inset) Structural formula of glycine.

The Free-Electron LASer in Hamburg (FLASH) at Deutsches Elektronen-Synchrotron DESY is capable of producing single-spike, self-amplified spontaneous emission (SASE) radiation pulses with a high degree of longitudinal coherence and a spectral bandwidth of Γ=0.37% at a central wavelength of $\lambda_{\text{FEL}}=4.55$ nm corresponding to an FEL photon energy of $E_{\text{FEL}}=272.7$ eV. The small spectral bandwidth of the fs pulses allows for resonant excitation of a particular core-shell transition in the glycine cation with high specificity while tracking the molecular dynamics with fs resolution. The single-spike operation at the FLASH facility is achieved by using a photoinjector laser with reduced pulse duration and by limiting the bunch charge compared to the typical SASE operation in order to facilitate the electron bunch compression.^22,23^ Running the electron gun with a pulse duration of 1 ps instead of 6.5 ps and bunch charges of 55 pC instead of 1 nC leads on average to 1.5 FEL spikes per photon pulse with Fourier-limited rms pulse duration of only (2.4 ± 0.2) fs, albeit at reduced x-ray pulse energies below 2 *μ*J. The pulse energies are measured with noninvasive gas monitoring detectors, which count the number of electrons generated in a low-density gas target on a pulse-to-pulse basis with an accuracy of 10%.^24^ Single-spike lasing at FLASH has been fully characterized for a central wavelength of 7 nm^25^ and has been used successfully for time-resolved FEL experiments at wavelengths down to 4.5 nm.^26,27^ Up to 500 photon pulses with 1 *μ*s spacing are grouped in a pulse train of around 0.5 ms total length. The nearly collimated FEL beam is focused by a nickel-coated toroidal mirror with a mirror surface of $25\times 25\;{\text{mm}}^{2}$ and 5.7 m focal length onto the molecular glycine beam. Assuming a TEM_00_ mode, the Rayleigh length and beam waist radius are 291 mm and 20 μm, respectively. The toroidal mirror is mounted on a hexapod that allows it to move the toroid with nm and μRad precision. This facilitates exact steering of the FEL beam through the detection chamber despite the 5.7 m long lever arm as well as matching the nominal 8° incidence angle of the toroidal mirror to prevent any astigmatism. A schematic overview of the experimental setup is shown in Fig. 1.

The electrons and ions generated in the interaction of the ultrashort FEL pulses with glycine molecules are detected simultaneously in opposite directions. In total, 13 electrostatic potentials at the ion TOF and the electron MBES can be applied to guide the particles as indicated in Fig. 1. Retardation voltages (Ret 1–4) can be used to discard low energy electrons and fine-tune the energy resolution of the remaining electrons. The ion drift tube needs to be quite short to allow for collection of the heavy and slow ions before the arrival of the next FEL pulse, i.e., keeping their time-of-flight below 1 *μ*s. This, however, reduces the mass-to-charge resolution. The magnetic field of the permanent magnet in the interaction zone is 400 mT, and the electron drift tubes are wrapped in a solenoid to form a homogeneous magnetic field of 8 mT, which yields a kinetic energy resolution of $\frac{\Delta E}{E}=2\%$. Both the electron and the ion side use double multichannel plates (MCPs) in a chevron arrangement. The initial MCP signals are fed into conventional pre- and output amplifiers before being further analyzed by a time-to-digital converter and recorded. The positions of the detector and electrodes of the MBES are fixed, whereas the positions of the interaction volume given by the FEL focus and the capillary position, the ion TOF spectrometer assembly, and the permanent magnet of the MBES can be adjusted.

A key component of the present experimental setup is the split-and-delay unit (SDU) based on interleaved reflective gratings.^28–30^ This device is located in front of the focusing mirror and has been described in detail elsewhere.^31^ Briefly, given the absence of transmissive beam splitters in the soft x-ray spectral range, splitting one FEL pulse into two time-delayed pulse replicas (pump and probe) can be achieved by two split half-mirrors with a relative longitudinal displacement and illuminated at grazing incidence. However, the two beams then need to be re-overlapped under an angle in the focus, which results in tilted wavefronts, thus averaging of their relative phase in the focal volume. Furthermore, large Mach–Zehnder SDUs that are usually used at FELs require several x-ray optics compromising stability and overall transmission of the device. Alternatively, using two interleaved reflective gratings in the present setup naturally produces two collinear pulses in a single reflection of each beam path. The generated pulse pair exhibits a constant phase difference across the beam profile with equal 1:1 intensity sharing and robust spatial overlap. One of these nickel-coated grating mirrors is fixed, while the second one can be vertically and horizontally pivoted in order to planarly align the two mirrors with nanometer precision under a fixed grazing incidence angle of 8°. The movable grating mirror can also be linearly displaced in the direction of the surface normal to enable the delay of one of the two pulse replicas. The 3D position of the movable mirror is monitored by means of optical laser and white-light interferometry (WLI) for absolute calibration of the FEL pump–FEL probe delays. Furthermore, the recorded data are used in an active feedback stabilization loop based on a field-programmable gate array while the delay is scanned. Thereby, the compact instrument minimizes temporal jitter and allows for time-resolved soft x-ray pump-probe electron and ion spectroscopy with a single-digit attosecond precision and a maximum useful scan length of about 1 ps time delay.^32^

## III. RESULTS AND DISCUSSION

### A. Time-resolved Auger electron spectroscopy upon resonant x-ray absorption

Important insight about the charge-induced chemical dynamics in glycine is gained from electron spectra taken as a function of x-ray-pump–x-ray-probe delay. Nonetheless, one has to keep in mind that high-energy FEL pump photons open-up various energy absorption and energy redistribution channels by generating electronically excited many-body states in the glycine cation.^33^ Thus, the main challenge of the present study is to discriminate those in order to observe the subsequent chemical dynamics upon photoionization of a particular inner-valence orbital. Glycine has 40 valence electrons occupying 20 closed-shell molecular orbitals (MOs) for which the first 17 binding energies are listed in Table I. The notations a′ and a ″ denote in- and out-of-plane orbitals. The a′ orbitals can comprise of *σ* and/or *π* symmetry MOs, whereas a ″ only comprises of *π* symmetry MOs. The discrimination between different valence ionization and subsequent relaxation channels is achieved by detecting the generated electrons and ions simultaneously shot-by-shot and by looking at electron–electron coincidences and electron-ion correlations. In the following, we discuss the overall coherent dynamics probed with time-resolved Auger electron and ion spectroscopy.

**TABLE I.** Experimental binding energies (BE) of glycine orbitals (conformer Gly I) in eV. a′ and a ″ denote different orbital orientations (in-plane and out-of-plane). O_C_ belongs to the carbonyl group (C = O), while O_H_ belongs to the carboxyl group (C–OH).

Orbital	BE
16a′ ( $n_{N})$ (HOMO)	10.0 ^a^
15a′ ( $n_{O})$	11.1 ^a^
4a ″ ( $\pi_{\text{OO}})$	12.2 ^a^
3a ″	13.6 ^a^
14a′	14.4 ^a^
13a′	15.0 ^a^
2a ″	15.6 ^a^
12a′	16.6 ^a^
11a′	16.9 ^a^
1a ″	17.6 ^a^
10a' (C_*α*_ 2s)	20.2 ^a^
9a′ (C 2s)	23.2 ^a^
8a′ (N 2s)	28.3 ^b^
7a′ (O_C_ 2s)	32.3 ^b^
6a′ (O_H_ 2s)	34.3 ^b^
5a′ (C_*α*_ 1s)	292.5^c^
4a′ (C 1s)	295.0^*c*^

^a^ Reference 34.

^b^ Reference 17.

^c^ Reference 35.

**FIG. 2.**
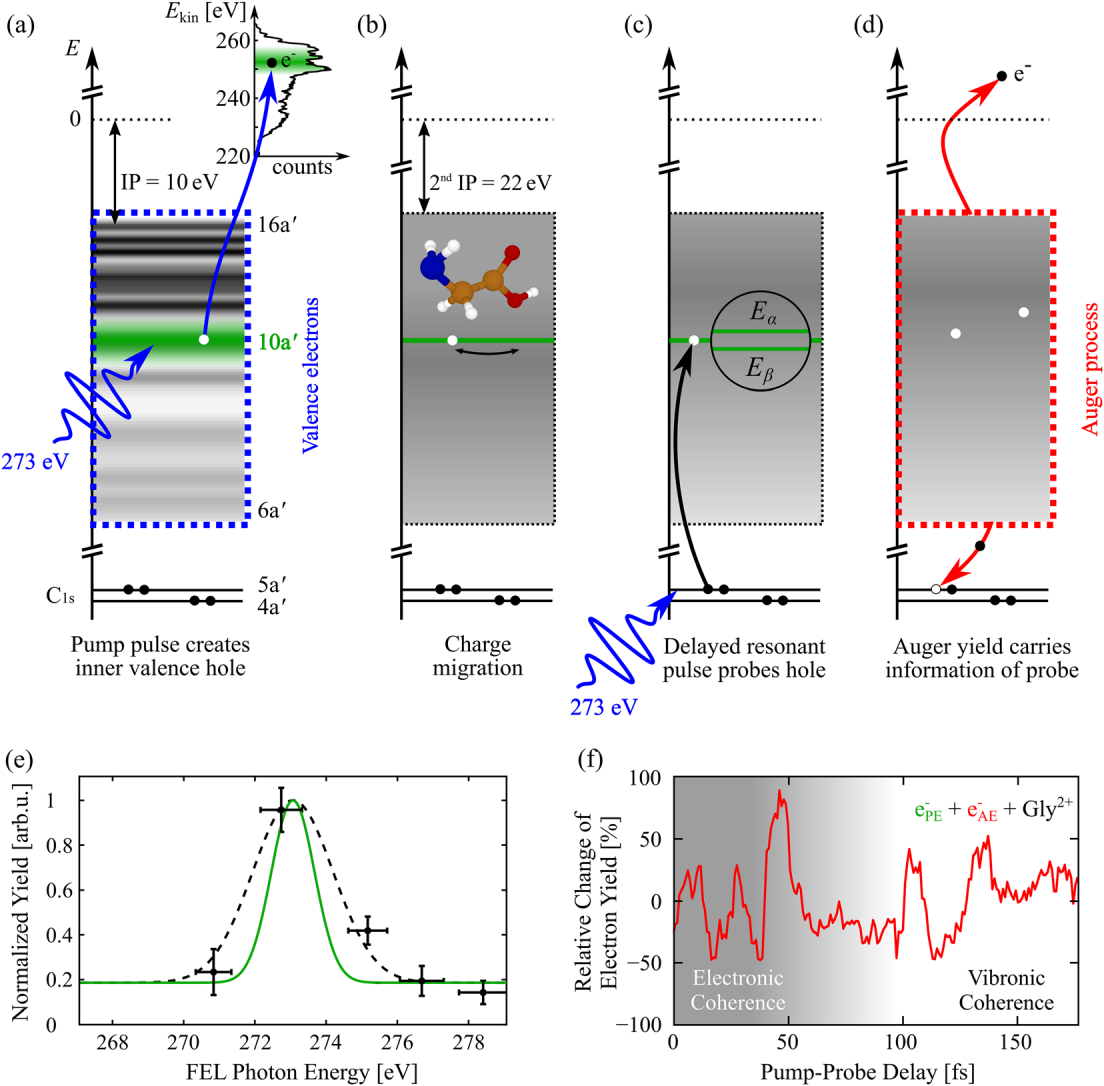
A single-color pump-probe scheme is applied to track charge-induced chemical dynamics initiated by photoelectron emission from the 10a′ molecular orbital. The total cascade involves several different processes: (a) pump-induced photoionization, (b) charge migration triggered by coherent population of ionized states, (c) resonant carbon 1s core-hole excitation, and (d) Auger decay. The individual steps are depicted as a sequence of energy diagrams. The density of states is indicated by using the experimental data reproduced with permission from Plekan *et al.*, J. Phys. Chem. A **111**, 10998–11005 (2007). Copyright 2007 American Chemical Society. (e) Two-electron coincidences plotted as a function of FEL photon energy with one electron being detected at the kinetic energy corresponding to valence ionization of 10a′. The resonance is fitted with a Gaussian envelope (black dashed line) and deconvoluted (green solid line) with respect to the spectral FEL bandwidth. (f) Relative change of the detected electron yield correlated with the generation of a Gly^2+^ parent ion and recorded together with an electron at the kinetic energy corresponding to valence ionization of 10a′ and an Auger electron as a function of x-ray pump-probe delay in 1 fs steps. The first ∼30–40 fs are attributed to an electronic coherence, while at later delays, vibronic coupling comes into play. The figure is adapted from Ref. 5.

The pump pulse with a central photon energy of 273 eV ionizes the glycine molecule, marking Δt=0. The kinetic energy of the outgoing photoelectron provides information about the involved MOs. In the applied experimental scheme depicted in Figs. 2(a)–2(d), events featuring electrons with $E_{\text{kin}}=(253\;\pm \;\Gamma_{\text{FEL}})$ eV, where $\Gamma_{\text{FEL}}=1.0$ eV is the FEL's bandwidth, from the 10a′ orbital are of interest. The 10a′ orbital spans nearly the full molecular backbone, and in consequence, the transient local electron–hole density moves to the same extent,^5^ thus making this orbital an excellent candidate for the study of charge-induced chemical dynamics involving geometric changes. The prepared electron–hole state undergoes oscillatory charge migration according to its effective and conformer-dependent level splitting of E≈0.2 eV mediated by electron correlations.^36,37^ The two distinct levels indicated in Fig. 2(c) for simplicity are both comprised of ≈50% of a pure inner valence hole state (1h) character and ≈50% of a series of two-holes-one-particle (2h1p) configurations.^35^ The superposition of theses cationic eigenstates possesses a high degree of electronic coherence on the order of 95% according to theory.^5^ If the electronic wave packet survives until after the variable time delay Δt, and the pure 1h state is localized in the vicinity of the C_*α*_ nucleus again, the 273 eV probe pulse will resonantly excite a C_*α*_ 1s electron into the 10a′ vacancy, allowing for subsequent Auger decay and emission of an Auger electron. The resonance of the probe-induced Auger decay as a function of photon energy was observed by counting the 10a′ photoelectron–Auger electron coincidences falling into the characteristic kinetic energy detection windows as shown in Fig. 2(e). This detection scheme selectively addresses coherent dynamics involving the 10a′ orbital. Other processes, which are certainly possible, do not contribute to the electron–electron coincidence data and, therefore, do not affect the interpretation of the data. In these measurements, the FEL photon energy was tuned in the range between 269 and 281 eV,^38^ which is specifically chosen to stay beneath the carbon K-edge (284.2 eV) as well as the nitrogen and oxygen K-edge (410 eV, 543 eV).^39,40^ Note that the molecule has two C1s orbitals, 4a′ and 5a′ with an energy difference of 2.9 eV, which is somewhat larger than the spectral bandwidth of the FEL. A coherent superposition of these states in the resonant absorption process is not possible with the limited x-ray pulse bandwidth. Also note that the corresponding time period of 1.4 fs for the 2.9 eV energy difference lies beyond the present time resolution of the pump-probe experiment.

The measured yield of Auger electrons vs pulse delay represents the oscillatory positive charge density with time period $T=19.6_{-1.4}^{+1.5}$ fs, since the 5a′ → 10a′ transition will more likely occur, the closer the transient hole is to the corresponding C_*α*_ atom. With this scheme, we could characterize the birth, propagation, and fate of the electronic coherences in a kinematically complete recent experiment.^5^ In brief, we counted the multi-particle events and plotted the relative change of the detected electron yield correlated with the generation of a Gly^2+^ parent ion and recorded together with an electron at the kinetic energy corresponding to valence ionization of 10a′ as a function of pump-probe delay. The time-resolved Auger electron spectroscopy result is shown in Fig. 2(f). Advanced *ab initio* many-electron simulations using the time-dependent B-spline restricted correlation space–algebraic diagrammatic construction (ADC) simulation method^41–43^ allowed us to explain the detected coherent quantum evolution in terms of the electronic coherence at early times. Its dynamics is monitored for a period of 175 fs. An important observation for the present study on charge-induced chemical dynamics mediated by nuclear motion is an evolving modulation that implicate the coupling of electronic to vibronic coherence at longer time scales. The glycine cation comprises *N* = 10 atoms resulting in 3N−6=24 normal modes for nuclear motion. As we will see in the following, if the probe-induced Auger electron yield is evaluated with respect to specific dissociation products of the generated doubly charged mother ion, we will gain information on the dominant reactive vibronic coherences leading to bond cleavage.

### B. X-ray-induced photoion–photoion coincidence (PIPICO) map

If the doubly charged glycine ion formed by the probe pulse fragments, the two positive charges can be distributed equally (1:1) or unequally (0:2) on the fragmentation products, dependent on homo- or heterolytic bond fission and location of the second charge. The newly created cations or dications may dissociate further. Neutral fragments remain intact and cannot be detected in the present work. The whole fragmentation sequence is likely to happen on a timescale of a few hundred fs.^44^ Soft x-ray induced fragmentation of glycine molecules in the gas phase has been studied by Itälä *et al.* using synchrotron radiation and a multi-particle coincidence technique.^45^ In the synchrotron experiments, a detailed fragmentation analysis of the sample molecule into pairs of momentum correlated cations has been carried out. The authors observed that the most common fragmentation pathway of Gly^2+^ starts with a cleavage of the C–C_*α*_ bond creating CH_2_ ${\text{NH}}_{2}^{+}$ and COOH^+^, followed by further fragmentation into the cations summarized in Table II or neutral species thereof. Their work also shows another characteristic fragmentation pathway for glycine, which is the elimination of a neutral (rarer cationic) water molecule, especially when there exists a hydrogen bond between the amino and hydroxyl groups.

**TABLE II.** Cationic ionization fragments of glycine created by the Auger decay process of the carbon 1s core vacancies studied at a synchrotron.^45^

*m/q*	Fragment
1	H^+^
2	$H_{2}^{+}$
12	C^+^
13	CH^+^
14	N^+^, ${\text{CH}}_{2}^{+}$
16	O^+^, ${\text{NH}}_{2}^{+}$
17	OH^+^
18	H_2_O^+^
27	CNH^+^
28	$N_{2}^{+}$, CO^+^, ${\text{CNH}}_{2}^{+}$
28.5	CH_2_NHCO^2+^
29	COH^+^, ${\text{CNH}}_{3}^{+}$
29.5	CH_2_NH_2_COH^2+^
30	[CH_2_–NH_2_]^+^
32	$O_{2}^{+}$
42	C_2_ ${\text{OH}}_{2}^{+}$, C_2_ ${\text{NH}}_{4}^{+}$
44	${\text{CO}}_{2}^{+}$
45	CO_2_H^+^
46	HCOOH^+^ and (OH) ${}_{2}$ C^+^
57	CH_2_NHCO^+^

**FIG. 3.**
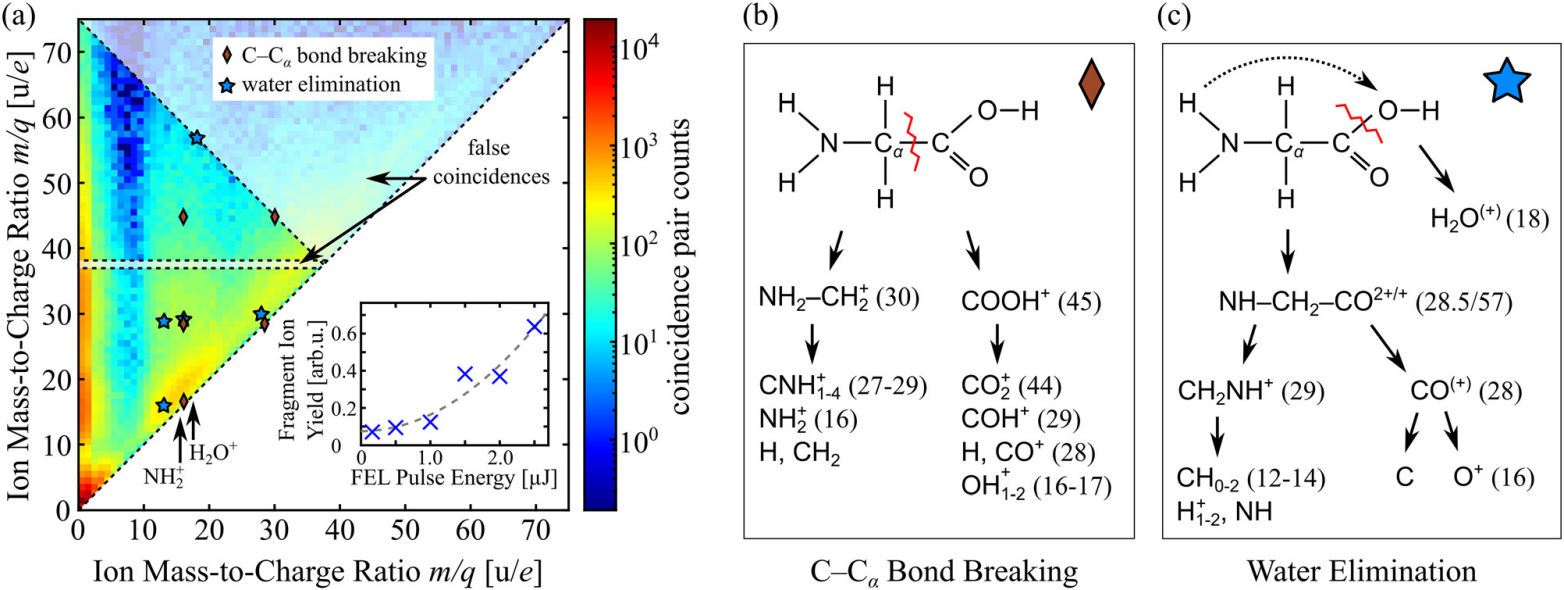
(a) Photoion–photoion coincidence map integrated over 175 fs pump-probe delay. Red diamonds indicate possible fragment pairs following the C–C_*α*_ bond breakage pathway, while blue stars indicate those from water elimination. Two notable regions of false coincidences have been marked (white shaded areas, see main text for details). Two different cationic fragments are indicated to give guidance. The total fragment ion yield recorded together with an electron at the kinetic energy corresponding to valence ionization of 10a′ depends quadratically on the FEL pulse energy as shown in the inset. This gives evidence to the 2-photon character of the underlying excitation process. (b) Glycine fragmentation pathways^45^ based on initial C–C_*α*_ bond breaking or (c) water elimination. Mass-to-charge ratios are given in brackets in u/*e* where applicable.

Before going into details on the soft x-ray induced chemical dynamics probed in our time-resolved study, we plot the time-integrated photoion–photoion coincidence (PIPICO) map in Fig. 3(a) for comparison with the synchrotron work as a starting point. This static information on all populated reactions channels is derived by integrating all detected coincidence ion pair counts over the 175 fs pump-probe scan presented in Fig. 2(f). Foremost, it shows that all photodissociation processes can be accompanied by H^+^ and $H_{2}^{+}$ losses. Secondary, the light to medium weight fragments form a pronounced off diagonal, showing that most fragmentation pathways lead finally to similar masses. However, also intermediate steps in the fragmentation processes are visible, which are vertically offset upward or horizontally offset to the right of their daughter ionic fragments in the PIPICO map. Very similar to the synchrotron work discussed above, the major fragmentation pathways include C–C_*α*_ bond breakage resulting in fragments with $\frac{m}{q}$ of (30, 45) and further splitting into (16, 45), (27–30, 28–29), (16, 28–29), and (16, 16–17) or minor variations as can be seen in Fig. 3(b). The decreasingly smaller fragment pairings exhibit exponentially increased yields. The second pathway of water elimination sketched in Fig. 3(c) produces the combinations (18, 57), (28, 29), (12–14, 28), (16, 29), or (12–14, 16). The doubly charged [NH–CH_2_–CO] ${}^{2+}$ cannot be present in the PIPICO map albeit as false coincidences. We note in passing that near-diagonal elements may also appear due to false coincidences. Furthermore, a coincidence of two cations with a combined mass larger than one glycine mass leads to false ion and thereby false electron coincidences. Similarly, any coincidence events including a dication and another charged ion result in false coincidences. The indicated region of false coincidences including Gly^2+^ is experimentally larger due to the limited ion TOF spectrometer resolution. For other dications, the false coincidences are more difficult to isolate because of overlap with singly charged cations.

From the time-integrated PIPICO-map shown in Fig. 3(a), one can conclude that the probe-induced core–shell transition, i.e., the resonant C_*α*_ 1s electron excitation into the 10a′ vacancy, mainly leads to fragmentation processes governed by the rupture of the C–C_*α*_ bond. It seems to involve the localization of the positive charges to the opposite sides of the C–C_*α*_ bond, which is reasonable from the viewpoint of Coulomb repulsion. However, it is important to note that the 10a′ inner-valence orbital (also in the dicationic state of glycine) is delocalized over the entire molecule as can be seen from the inset in Fig. 2(b). Therefore, the probe-induced ion fragment distribution is quantum mechanically determined by the Auger final states of Gly^2+^, not by classical Coulomb forces as pointed out already by Itälä *et al.*^45^ Furthermore, the resonant 5a′ → 10a′ transition between the electronic states as a function of pump-probe delay is accompanied by a transition of vibrational states. Note the vibronic coupling, which results in a change of the internuclear distance, is greatly enhanced in the vicinity of conical intersections or avoided crossings.^46^ In this case, the Born–Oppenheimer approximation fails, and the nuclear and electronic wave functions can no longer be separated. Indeed, energy spacings in the energy region of partial breakdown of the molecular orbital picture to which the 10a' states of the glycine cation belong are of the same order of magnitude as some of the vibrational quanta, and the two degrees of freedom are expected to strongly couple, resulting in quantum eigenstates represented by linear superpositions of electronic states dressed by vibrational excitations.^47^ Thus, the full electronic coherence initially brought to life by the few-fs ionization of glycine in the inner valence region discussed in Subsection III A is very likely of mixed electronic and vibrational character after a few oscillation periods. However, full characterization of this coherence theoretically within the highly computationally demanding inner-valence energy region is currently beyond reach and should be subject of future theoretical studies. We note in passing that vibronic coherence has been studied computationally by Mukamel and co-workers in the case of core ionization or excitation,^48–51^ where breakdown of the molecular orbital picture is not typical. From an experimental point of view, it is possible to further elaborate on particular vibronic couplings in the glycine cation, namely, those that lead to a particular chemical reaction, i.e., a particular dissociation product. As we will see in the following, from the evaluation of the long time span of coherent signal oscillations observed in the course of specific fragment formation, we can learn something on charge-induced chemical dynamics. Again, it is important to note that in our work, we probe experimentally, via electronic degrees of freedom, the cationic state coherence of the intact glycine molecule resulting from the inner-valence ionization prior to charge-induced fragmentation. We have verified that under our experimental conditions, the fragmentation of glycine molecules is in total a two-photon process. It comprises inner-valence ionization by the pump photon and core–shell excitation by the probe photon, which relaxes via Auger decay generating doubly charged parent ions. Accordingly, the recorded fragment ion yield shows a quadratic dependence of this reaction path on the FEL pulse energy. The corresponding data are plotted in the inset of Fig. 3(a) for zero time delay.

### C. Wavelet analysis of multi-particle correlations between electrons and fragment ions

**FIG. 4.**
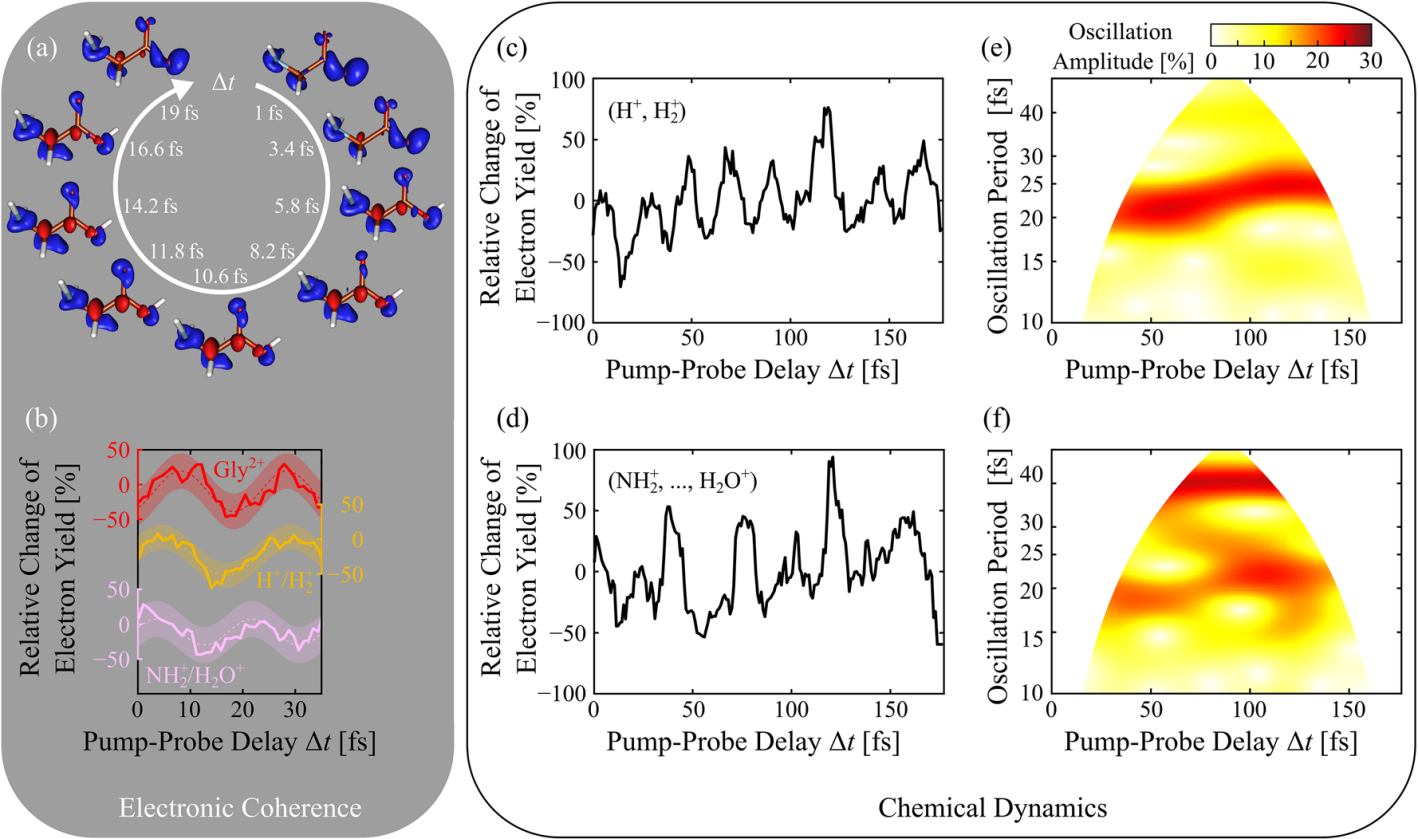
(a) *Ab initio* simulations showing the 10a′ electron density (red lobes) over a full oscillation period. (b) Relative change of the 10a′ photo- and Auger electron yield at early times in correlation with detected Gly^2+^, (H^+^, $H_{2}^{+}$), and (N $H_{2}^{+}$,…, H_2_O^+^) ions, respectively. A sinusoidal fit with 95% error bounds (shaded area) gives the time periods $19.6_{-1.4}^{+1.5}$, $23.9_{-1.1}^{+1.2}$, and $18.2_{-1.4}^{+1.7}$ fs and phases (−0.3 ± 0.1)π, (0.1 ± 0.1)π, and (0.1 ± 0.2)π for the respective electronic coherences. (c) Relative change of the 10a′ photo- and Auger electron yield in correlation with (H^+^, $H_{2}^{+}$) and (d) (N $H_{2}^{+}$,…, H_2_O^+^) for the full 175 fs measurement duration. (e) Continuous wavelet transform (CWT) of the recorded pump-probe signal in (c) showing a clear oscillation period in the range of 21–25 fs. (f) CWT of the signal in (d) showing a superposition of a 19–22 and a 40 fs time period.

The remaining question is whether the subsequent electron-nuclear dynamics mediating the dissociation of the dication—after the second ionization produced by the probe pulse—still allows one to disentangle the coherent dynamics observed in the cation. In particular, it is interesting to ask the question whether it is possible to extract different characteristics of the electronic coherence at early times depicted in Fig. 4(a) by correlating the recorded time-resolved electron spectra with the occurrence of specific fragments in the TOF mass spectra. Apart from the photoelectron–Auger electron–Gly^2+^ (three-particle) correlation, two further species of correlated cationic fragments show fingerprints of x-ray pump-induced coherent dynamics in the recorded three-particle correlation. These are H^+^ and $H_{2}^{+}$, respectively, as well as the group of N $H_{2}^{+}$, O^+^, OH^+^, and H_2_O^+^ that could not be resolved because of the limited mass-resolution of the short TOF spectrometer. Figure 4(b) shows the change of the time-dependent electron yield up to pump-probe delays of 35 fs for the two groups including the data related to the Gly^2+^ parent ion for comparison. All pump-probe traces use the same electron kinetic energy selection as before, i.e., the analysis is focused on glycine Auger electron emission and subsequent chemical dynamics following 10a′ photoionization.

It can be seen that the different fragmentation channels still monitor the charge (hole) migration in the spatially extended 10a' molecular orbital with a time period of ≈20 fs albeit showing slightly different relative phases. This experimental result supports the assumption that by measuring specific fragments associated with the dication as a function of the pump-probe delay, one can selectively probe electronic coherences associated with a few components of the electronic wave packet created initially by the pump pulse in the cation as predicted theoretically by Delgado *et al.*^52^ It seems that the energy gaps between the coherently coupled electronic states do not vary too much along the reaction coordinate during the first few tens of fs, allowing the electronic coherences to survive long enough to leave their signature in the time-resolved fragmentation spectra.

In order to fully unravel the impact of vibrational modes on the energetics and dynamics of the populated cationic states, further theoretical work is necessary. Recently, interesting results have been reported by Delgado *et al.*^52^ The fragmentation study evaluated the full-electron wave function including non-adiabatic effects and revealed early electron dynamics induced by attosecond pulses. A direct comparison is somewhat difficult, since in the present experiments, small-bandwidth, soft x-ray FEL pulses at 273 eV have been applied, whereas the theoretical work focused on broadband XUV photon pulse excitation typical for high-harmonic generation (HHG) sources. The calculations were performed for pulses centered at three different photon energy, 12, 16, and 20 eV, which result in much broader wave packets and, thus, shorter time periods of the induced coherences.

The complete ion-correlated XPS dataset spanning a 175-fs timescale for the two groups of fragments is shown in Figs. 4(c) and 4(d). At first glance, the data show pronounced oscillations also for long delays. In order to better understand the dynamic oscillation period and its amplitude evolution, a time-frequency distribution was produced using the continuous wavelet transform with Airy wavelets (see the Appendix and Ref. 53). The oscillation frequencies in the corresponding false color plots presented in Figs. 4(e) and 4(f) were converted to periods for better comparability. The so-called “cone of influence,” where part of the wavelet in time domain extends past the finite recorded experimental signal trace, is removed from each plot. Here, artificial edge effects disturb the frequency analysis at early times and, therefore, are not taken into account in the present analysis of charged-induced chemical dynamics for long delays in excess of 35 fs. Vibrational timescales for small molecules such as glycine are generally in the range of 10–100 fs for particular bonds and partial groups and up to 1 ps for intramolecular vibrations.^54^ Rotations of small moieties around bonds are significantly slower and carried out on timescales of 100–350 ps,^55^ which is far too slow to affect the present results. What follows is a brief discussion of potentially involved vibrational modes mediating charge-induced bond cleavage along different reaction coordinates albeit not yet substantiated by theoretical simulations of the induced chemical dynamics.

#### 1. H^+^ and $H_{2}^{+}$ PEPEPICOV yield

We assume that the relative change of the electron yield correlated with H^+^ and $H_{2}^{+}$ ion detection monitors part of the initial electronic coherence that couples to CH_2_ bending (*δ*) and wagging (*ω*) vibrational modes with time periods in the range of 22.8 and 23.1 fs, respectively.^56^ The detected protons and $H_{2}^{+}$ ions together with the Auger electron signal, which is particularly sensitive to the local C_*α*_ 1s probe pulse absorption (5a′ → 10a′), likely originate from the –C_*α*_H_2_–moiety of glycine. Here, the hole state localizes, and the resonant core-inner valence absorption can take place before any other fragmentation of the molecule sets in. In other words, the local soft x-ray probe pulse absorption into the 10a′ state at the –C_*α*_H_2_– moiety populates dissociative reaction channels of vibrationally excited glycine molecules. We are aware of the fact that the picture of hydrogen fragmentation from hot related vibrational modes, although physically reasonable, remains incomplete and ultimately requires a more detailed formulation. Basically, the many body wave packet dynamics shown in Fig. 4 is of mixed electronic and vibrational character. The full time-dependent characterization of its time-frequency spectrum theoretically within the highly computationally demanding inner-valence energy region is currently beyond reach and should be the subject of future theoretical studies.

#### 2. N $H_{2}^{+}$ and H_2_O^+^ PEPEPICOV yield

The same holds true for the observed chemical dynamics, which involves the generation of N $H_{2}^{+}$, O^+^, OH^+^, and H_2_O^+^ ionic fragments being even more complex. According to the wavelet analysis of the recorded data, it contains both a 20.6 fs and strong 39.4 fs vibronic component covering the first ∼130 fs. These fragments are products from both discussed fragmentation pathways (C–C_*α*_ bond breaking or water elimination, see Fig. 3), making the interpretation of the vibrational modes difficult. Slower, delayed oscillations can be generally attributed with intramolecular degrees of freedom for nuclear motion. According to experimental work by Rosado *et al.*,^56^ there exists a prevalent vibrational mode with a period of 41.7 fs mainly consisting of *ν*C–C stretching (45% contribution) and *ω*NH_2_ rotation (13%). Another vibrational mode with intermediate intensity consisting of *ω*NH_2_ (46%), *ν*C–C (17%), and *δ*NH_2_ (16%) has a period of 37.8 fs.^56^ The 20.6 fs oscillation shown in Fig. 4(f) likely does not relate to electronic coherences as it is present for over 130 fs but instead it might relate to NH_2_ bending (*δ*NH_2_ (71%), *ω*NH_2_ (24%)) with a period of 20.5 fs.^56^ The *ν*C = O stretch vibration has a similar period of 18.7 fs^56^ and might be enabled in this selective ion channel after the electronic decoherence time, requiring coherent vibronic coupling. Additional characteristic modes such as C–O stretching with 30.3 fs, C–N stretching plus C–C vibrations corresponding to 32.2 fs,^57^ and further C–C stretching modes with periods of 24.1^56^ and 26.4 fs^58^ might also play a role in the recorded time-dependent fragmentation pattern. However, their relative contribution cannot be extracted from the present dataset.

We would like to emphasize that all values taken from Ref. 56 are reported for the neutral glycine molecule. Therefore, the data interpretation striving to pinpoint the driving vibrational modes in the cation that mediate bond cleavage along different reaction coordinates can only be regarded as a first attempt to shine light on the charged-induced chemical dynamics at work in this many-body quantum system. Furthermore, the involvement of NH_2_ to a lesser degree O^+^, OH^+^, and H_2_O^+^ in both the recorded ion yield and the related vibrational modes implies that the oscillatory yield pattern is a result of protonation likelihood based on the oscillatory intramolecular –H ⋯ O– proximity. It has yet to be resolved whether each of the two main oscillations (20.6 and 39.4 fs) can be attributed to a particular fragment. It is so far also unclear what role the resonant C_*α*_ 1s to inner valence excitation (5a′ → 10a′), for which the Auger electron channel is sensitive, plays in the protonation process. In any case, we are convinced that the presented experimental findings will prove beneficial for the development of theoretical treatments of the complex interplay between electronic states and nuclear degrees of freedom.

## IV. SUMMARY AND OUTLOOK

Time-resolved Auger electron spectroscopy in a single-color, x-ray pump-probe scheme was applied to study charge-induced chemical dynamics in the cation of the amino acid glycine (Gly^+^). The breaking of covalent bonds in the molecule is triggered by inner-valence photoionization with few-fs pulses at a photon energy of 273 eV. This pump photon energy was chosen because it opens an orbital-selective and element-specific detection window to probe charge dynamics exclusively in the 10a′ molecular orbital. The discrimination is based on the probe-induced, resonant core-shell electron transition (5a′ → 10a′) followed by Auger decay. Coincidence detection and covariance mapping of electrons and ions generated in the interaction of Gly^+^ with the time delayed fs FEL probe pulses allowed to trace the life cycle of the electronic wave packet including its birth, propagation, and fate. By correlating the recorded x-ray photoelectron spectroscopic data to specific fragment ions as a function of time delay, we monitored how the initially prepared pure electronic wave packet of the spatially extended 10a′molecular orbital couples to specific vibrational modes propagating along selected dissociation pathways, while the charge-induced chemical dynamics proceeds in the parent ion.

The key result is that different fragmentation channels still monitor the initial charge migration in the 10a′ molecular orbital at early times with a time period of ≈20 fs albeit showing slightly different relative phases. This observation indicates that by measuring specific fragments associated with the dication as a function of the pump-probe delay, one can selectively probe electronic coherences at early times associated with a few components of the broad electronic wave packet induced by the pump pulse in the cation. After a few oscillations, strong vibronic coupling sets in because the energy spacings in the energy region of partial breakdown of the molecular orbital picture to which the 10a′ states belong are of similar magnitude as some of the vibrational quanta. It results in a complex interplay between electronic states and nuclear degrees of freedom. Even at short timescale vibronic coherence can play a role, and further theoretical work is necessary to disentangle (if possible) the impact of electron and vibronic coherence.

Obviously, the initial ionization starts multiple dynamical pathways, which are driven by the electronic 10a′ superposition state dressed by multiple vibrational modes. From the experimental data, we conclude that the transient electronic signature corresponding to specific channels (H^+^, $H_{2}^{+}$) and (N $H_{2}^{+}$,…, H_2_O^+^) is already subtlety different, and, for instance, if we could fully resolve the Auger spectrum (down to vibrational substructure) or could measure the XPS at high resolution, we assume that we would be able to identify these two distinct channels emerging after ≈35 fs also spectroscopically. Nonetheless, this subtle difference is seen in the small phase and time period differences of the electronic coherences observed in Fig. 4(b). Taking together the electronic and nuclear system, these parallel dynamical pathways evolve into the propensity for different fragment patterns following the probe step. In other words, the selected fragment ion changes the contributions of the vibrational modes to the specific observed pathway.

The time-frequency spectra of the many-body quantum mechanical wave packets represented by coherent superpositions of electronic states dressed by vibrational excitations have been measured for the first time along different reaction coordinates in the cation. We could show that the observed coherences reveal rich information on the many-body quantum system including ultrafast decay and site-specific couplings that differ in phase. The presented experimental results provide a strong incentive for further development of theoretical tools to approach these important aspects of many-body quantum dynamics.

In order to retrieve absolute phase information of the excited coherences from the experimental data, more sophisticated ultrafast methods are necessary, which rely on nonlinear interferometric measurements.^59^ Wavepacket interferometry and coherent multidimensional spectroscopy are well-established methods to probe the structure and dynamics of quantum systems with high time resolution and wavelengths ranging from the ultraviolet to the far infrared and beyond.^60^ The transfer of these methods toward XUV and soft x-ray photon energies is, of course, highly desirable.^61^ However, it imposes quite some challenges to the experimentalists due to the required ultra-high timing and phase stability of the instrumentation,^62^ and only a few examples have been reported so far.^63–66^ In any case, it can be safely concluded that the pioneering experimental and theoretical works in the field of ultrafast x-ray atomic and molecular physics in the past decade open up new exciting possibilities to study many-body quantum effects in electron and nuclear dynamics.^67^

## Data Availability

The data that support the findings of this study are available from the corresponding author upon reasonable request.

## APPENDIX: WAVELET ANALYSIS

In general, a wavelet analysis allows to study the amplitude evolution of a non-stationary signal at scaling frequencies.^53^ A wavelet $\psi (t)=g(t)e^{i\omega t}$ is a modulated signal with frequency *ω* convoluted with an envelope *g*(*t*). The classification and naming of wavelets refer to the shape of the envelope. The generalized Morse wavelets encompass a wide selection of wavelets by introducing the shaping parameters *β* and *γ* describing the width of the envelope in frequency and time domain. With *γ* = 3, so-called Airy wavelets, the most Gaussian-like envelope shape can be achieved as well as the symmetry in frequency domain maximized. The generalized Morse wavelets $\Psi_{\beta ,\gamma }(\omega )$ in frequency domain are given by Lilly and Olhede^68^ as

$$\Psi_{\beta ,\gamma }(\omega )=\int_{-\infty }^{\infty }\psi_{\beta ,\gamma }(t)\;e^{-i\omega t}\;dt=H(\omega )\;c_{\beta ,\gamma }\;\omega^{\beta }e^{-\omega^{\gamma }},$$
(A1)

where $c_{\beta ,\gamma }$ is a normalization constant and the Heaviside function H(ω) ensures that $\Psi_{\beta ,\gamma }(\omega )=0$ for ω<0. We use a time-bandwidth product of βγ=60 and 48 voices per octave. The boundary where part of the wavelet in time domain extends past the finite signal is called “cone of influence” (COI). The COI is chosen as the points where the autocorrelation magnitude of the respective wavelet decays by $\frac{1}{e}$.^69^
